# Noninvasive Free Flap Monitoring Using Eulerian Video Magnification

**DOI:** 10.1155/2016/9471696

**Published:** 2016-03-22

**Authors:** Yuan Fang Liu, Christopher Vuong, Paul Charles Walker, Nathaniel Ray Peterson, Jared Christian Inman, Pedro Alcantara Andrade Filho, Steve Choon-Sung Lee

**Affiliations:** Department of Otolaryngology-Head and Neck Surgery, Loma Linda University Medical Center, Loma Linda, CA, USA

## Abstract

Eulerian Video Magnification (EVM) can enhance subtle changes in videos to reveal what was once invisible to the naked eye. In this proof of concept study, we investigated using EVM as a novel form of free flap monitoring. Free flaps with skin paddles were filmed in the operating room with manipulation of their pedicles. In a representative 77-year-old female who received a latissimus dorsi-serratus-rib composite free flap, EVM was able to detect blockage of arterial or venous supply instantaneously, providing a visible representation through degree of color change in videos. EVM has the potential to serve as a powerful free flap monitoring tool with the benefit of being noninvasive, sensitive, easy-to-use, and nearly cost-free.

## 1. Introduction

Despite modern rates of success in free flap reconstruction surgery of greater than 95%, accurate and timely identification of problems in the postoperative period remains critical for flap exploration and salvage [1–5]. Studies have repeatedly shown that the timing of detection of vascular compromise is one of the most important factors in determining success of salvage, such that flap survival in take-backs decreases with increasing time postoperatively and is negligible beyond 72 hours [6–8]. Thus, careful monitoring and early recognition of flap complications are crucial.

An ideal monitoring system should be accurate, sensitive, consistent, noninvasive, easy-to-use, and cost-effective. Existing free flap monitoring modalities vary in invasiveness, efficacy, and difficulty of application. No ideal system has been described or widely accepted. Eulerian Video Magnification (EVM) is a way of enhancing videos to reveal nearly invisible changes in color or motion. It was developed by the Massachusetts Institute of Technology Computer Science and Artificial Intelligence Laboratory (MIT CSAIL) as an algorithm to filter out temporal variations in color or motion and amplify them to make subtle changes in videos more apparent [9]. We sought to explore the ability of EVM to detect perfusion changes in free flaps.

## 2. Case Presentation

A 77-year-old female with a history of hypertension and hypothyroidism, status-post radiation failure, underwent radical excision of a buccal squamous cell carcinoma and received a latissimus dorsi-serratus anterior-rib composite free flap. The free flap had an ischemia time of 2 hours and 43 minutes. The free flap thoracodorsal artery was anastomosed to the right internal maxillary artery, and the accompanying vein was anastomosed to a branch of the right internal jugular vein. Recordings were taken of the free flap before inset, with the artery clamped, the vein clamped, or neither vessel clamped. The patient's blood pressure was 90/55 during filming.

Filming was performed using a Sony *α*55 DSLR camera with an 18–55 mm zoom lens at 1440 × 1080 pixels' resolution. Recordings were made at 30 frames per second in MP4 format. A tripod was used, and manual focus was used in lieu of autofocus to prevent image blurriness caused by focus changes with minor movements.

EVM was applied to the videos using the Videoscope application available at https://people.csail.mit.edu/mrub/vidmag/ in order to amplify color changes. In basic terms, what EVM does is that it looks at a fixed position in a video (deconstructs the video into different spatial frequency bands) and tracks the change in color over time. It then creates a new video by amplifying (by a desired factor) the changes in color at this position, thereby creating an overall effect of exaggerated color change or motion in the video. Technical details of the algorithm are outside the scope of this paper but can be found in Wu et al.'s research [9].

The amplified frequency range was chosen to be the heart rate +/− 0.1 Hz (1.2–1.4 Hz). The filter type was set to “ideal.” The chrominance scale (0-1) was set to 1. The spatial cutoff wavelength was set to 4. The above parameters were chosen based on trial and error to achieve subjective optimal results. Details on the modifiable parameters of the EVM algorithm can also be found in Wu et al.'s study on EVM and the website above [9].

**Figure 1:**
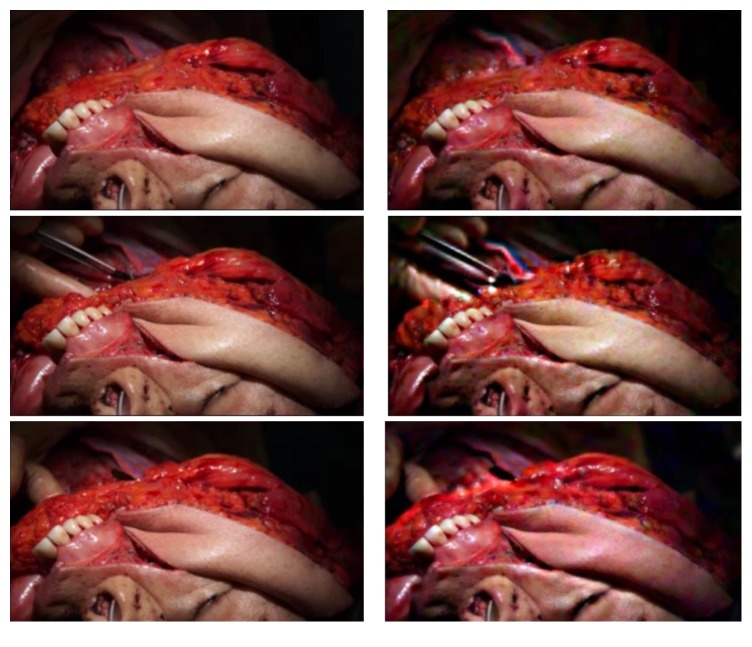
Flaps at peak outflow and inflow of blood with Eulerian Video Magnification at 200x. Rows top to bottom: no clamp, artery clamped only, and vein clamped only. Left: outflow, right: inflow. Notice the difference in color between the gingiva and skin paddle.

The free flap transformed with EVM at 200x magnification is shown in Video 1 (Supplementary Material) in Supplementary Material available online at http://dx.doi.org/10.1155/2016/9471696. When the artery was clamped, the free flap skin paddle showed a subjective, immediate decrease in pink-red tinge with inflow of blood as compared to the surrounding skin and gingiva and as compared to itself when neither vessel was clamped, as shown in Video 2 (Supplementary Material). Likewise, when the vein was clamped, the skin paddle was subjectively more pink-red tinged instantaneously as compared to the surrounding tissues and as compared to itself when neither vessel was clamped, as shown in Video 3 (Supplementary Material). Screenshots of the flaps at peak blood outflow and inflow are shown in Figure 1.

## 3. Discussion

Current free flap monitoring methods include physical exam (skin color, turgor, temperature, and capillary refill), surface temperature recording, external Doppler, implantable Doppler, color duplex sonography, laser Doppler flowmetry, infrared thermography, near-infrared spectroscopy, white light spectroscopy, and microdialysis [10–14]. Perhaps the closest to ideal modality is near-infrared spectroscopy, which uses optical spectrometry to measure tissue oxygenation [15]. It has been shown to be highly sensitive, specific, and reliable [10, 16, 17]. However, its drawbacks include the need for frequent probe repositioning and the price of the system, which requires a reusable console costing about 20,000 dollars and disposable sensors costing about 650 dollars each [10, 12, 17]. Near-infrared spectroscopy studies in animals have shown that arterial and venous occlusions were detectable within seconds after occlusion [15, 18]. EVM seems to be at least comparable, if not more sensitive than near-infrared spectroscopy.

Free flap monitoring through EVM can reveal changes in perfusion without physical contact with the flap or change in patient management and with negligible added expense. With access to the free EVM software online, any person using a video camera can perform free flap monitoring. A member of the team which created the algorithm stated that a real-time version, which can potentially be downloaded as an application on a smartphone, is under development. This would allow instantaneous assessment of flap perfusion or continuous free flap monitoring with a bedside video camera.

Several analyses that must be performed before EVM can be used clinically. Objective measures must be established to correlate the degree of color change to the degree of arterial or venous occlusion. Also, blood pressure changes and how they affect free flap perfusion and color change on EVM must be assessed. Despite the lack of standard values, EVM can still be used to visualize changes in perfusion if an initial video is taken after anastomosis and inset to use as a baseline. In this way, EVM may serve as an adjunct to other well-established methods of flap monitoring.

We found that filming of flaps after dissection of the flap to be transferred, but before ligation of the pedicle from the donor site, produced inferior results compared to filming after anastomosis to the receiving site in the head and neck. We hypothesize that this is due to greater perfusion of the head and neck region, such that blood flow through the face and mucosal surfaces is more apparent and can be more easily contrasted to the free flap blood flow. Another possibility is that the feeding artery and receiving vein within the head and neck provide better perfusion to the free flap than its native vessels. Furthermore, issues with reperfusion hyperemia cannot be discounted.

A current impediment to using EVM as a free flap monitoring tool is the need for the subject and the video camera to be relatively motionless, because motion artifact can be confused with changes in color indicating perfusion alterations. However, software adaptations may be able to filter out nonpatterned motion (as opposed to heartbeats) to diminish or eliminate motion artifact. Also, adequate lighting of the free flap must be available such that video recordings are able to detect changes in color. Research is needed to establish standard parameters for and to verify the accuracy and reliability of EVM. Nevertheless, this initial proof of concept study may serve as an impetus for further research by highlighting the potential of EVM as a noninvasive, sensitive, cost-effective, easy-to-use, and reproducible flap monitoring tool.

## Supplementary Material

Video 1. Free flap without (left) and with (right) Eulerian Video Magnification at 200 times magnification with no vessel manipulation.
Video 2. Free flap without (left) and with (right) Eulerian Video Magnification at 200 times magnification with artery clamped only.
Video 3. Free flap without (left) and with (right) Eulerian Video Magnification at 200 times magnification with vein clamped only.
